# Neighborhood factors and triple negative breast cancer: The role of cumulative exposure to area‐level risk factors

**DOI:** 10.1002/cam4.5808

**Published:** 2023-03-14

**Authors:** Scott D. Siegel, Madeline M. Brooks, Jesse D. Berman, Shannon M. Lynch, Jennifer Sims‐Mourtada, Zachary T. Schug, Frank C. Curriero

**Affiliations:** ^1^ Institute for Research on Equity & Community Health, Christiana Care Health System Newark Delaware USA; ^2^ Helen F. Graham Cancer Center & Research Institute, Christiana Care Health System Newark Delaware USA; ^3^ Division of Environmental Health Sciences University of Minnesota School of Public Health Minneapolis Minnesota USA; ^4^ Cancer Prevention and Control, Fox Chase Cancer Center Philadelphia Pennsylvania USA; ^5^ The Wistar Institute Cancer Center Philadelphia Pennsylvania USA; ^6^ Department of Epidemiology, Johns Hopkins School of Public Health John Hopkins Spatial Science for Public Health Center Baltimore Maryland USA

**Keywords:** cumulative exposure, disparity, environmental hazards, neighborhood, segregation, triple negative breast cancer

## Abstract

**Background:**

Despite similar incidence rates among Black and White women, breast cancer mortality rates are 40% higher among Black women. More than half of the racial difference in breast cancer mortality can be attributed to triple negative breast cancer (TNBC), an aggressive subtype of invasive breast cancer that disproportionately affects Black women. Recent research has implicated neighborhood conditions in the etiology of TNBC. This study investigated the relationship between cumulative neighborhood‐level exposures and TNBC risk.

**Methods:**

This single‐institution retrospective study was conducted on a cohort of 3316 breast cancer cases from New Castle County, Delaware (from 2012 to 2020), an area of the country with elevated TNBC rates. Cases were stratified into TNBC and “Non‐TNBC” diagnosis and geocoded by residential address. Neighborhood exposures included census tract‐level measures of unhealthy alcohol use, metabolic dysfunction, breastfeeding, and environmental hazards. An overall cumulative risk score was calculated based on tract‐level exposures.

**Results:**

Univariate analyses showed each tract‐level exposure was associated with greater TNBC odds. In multivariate analyses that controlled for patient‐level race and age, tract‐level exposures were not associated with TNBC odds. However, in a second multivariate model that included patient‐level variables and considered tract‐level risk factors as a cumulative exposure risk score, each one unit increase in cumulative exposure was significantly associated with a 10% increase in TNBC odds. Higher cumulative exposure risk scores were found in census tracts with relatively high proportions of Black residents.

**Conclusions:**

Cumulative exposure to neighborhood‐level risk factors that disproportionately affect Black communities was associated with greater TNBC risk.

## Graphical abstract

In a multilevel model that adjusted for patient age at diagnosis and race, a one‐unit increase in the cumulative exposure to census tract‐level measures of unhealthy alcohol use, metabolic dysfunction, breastfeeding, and environmental hazards was associated with a 10% increase in the odds of triple negative breast cancer (TNBC) relative to other subtypes of invasive breast cancer. Higher cumulative exposure risk scores were found in census tracts with relatively high proportions of Black residents, which may help to explain racial disparities in breast cancer mortality.

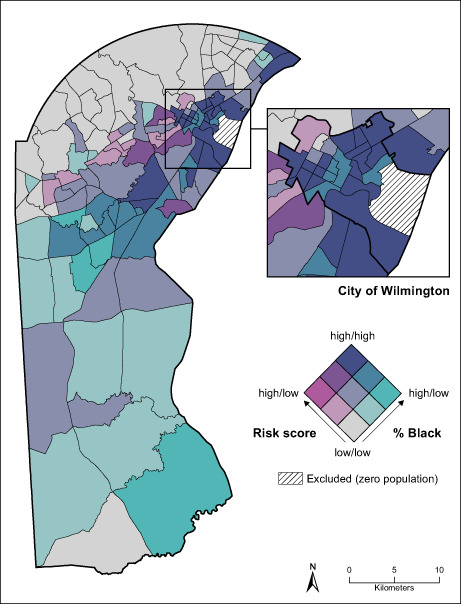

## 1 INTRODUCTION

Black women disproportionately bear the burdens of breast cancer in the US. Breast cancer is the leading cause of both cancer incidence and mortality among Black women,^1^ with 41% higher breast cancer mortality among Black women relative to White women despite comparable incidence.^1^ Multiple structural barriers to healthcare access have been linked to this racial disparity. Relative to White women, Black women are less likely to receive high‐quality screening,^2^, ^3^, ^4^ be diagnosed at an early stage,^5^, ^6^, ^7^, ^8^ receive a diagnosis and begin treatment without delay,^9^, ^10^, ^11^, ^12^ and receive guideline‐concordant care.^13^, ^14^, ^15^, ^16^ However, access to care does not fully account for the racial disparity in breast cancer mortality.^17^, ^18^

Tumor biology may underpin Black‐White differences in breast cancer mortality not solely attributable to healthcare access. In particular, triple negative breast cancer (TNBC) is an aggressive subtype of invasive breast cancer that is twice as prevalent among Black women.^19^, ^20^ TNBC is negative for the estrogen receptor (ER), progesterone receptor (PR), and human epidermal growth factor 2 (HER2), and therefore does not respond to the targeted therapies that have largely explained the improvements in breast cancer outcomes in recent decades.^21^, ^22^ Given the poorer outcomes associated with TNBC and the racial differences in prevalence, TNBC accounts for more than half of the Black‐White difference in breast cancer deaths.^23^ Thus, until more effective treatments are developed for TNBC, and healthcare access disparities are addressed, primary and secondary prevention may offer the best prospect for closing racial disparities.^24^

Efforts to improve the prevention of TNBC have been stymied by a lack of progress in identifying risk factors that have been validated among Black women.^25^ To date, a relatively small set of patient‐level characteristics beyond race have been consistently linked to TNBC risk, including younger age, reproductive factors (e.g., breastfeeding), and metabolic dysfunction.^20^, ^26^ Genetic factors have also been linked to the higher rates of TNBC observed among Black women in the US, particularly for those of western Sub‐Saharan African ancestry.^22^ However, shifting TNBC incidence rates over time and by race and geographic region implicate gene x environment interactions.^27^ Indeed, two recent high‐quality multilevel studies that adjusted for patient socioeconomic status (SES)^28^, ^29^ have added to a growing body of research^30^, ^31^, ^32^, ^33^ establishing a link between neighborhood SES and TNBC risk. Better understanding the apparent link between contextual exposures and TNBC risk may accelerate the identification of additional risk factors and improve prevention.

Building on these efforts, we previously considered the role of residential segregation for TNBC incidence. Given the well‐documented and pervasive pattern of residential segregation in the US^34^, ^35^ and its implications for cancer outcomes,^36^, ^37^, ^38^, ^39^, ^40^ we drew on several conceptual models that consider multilevel influences on health disparities^41^, ^42^, ^43^ and hypothesized that residents of predominantly Black neighborhoods are disparately exposed to TNBC risk factors. In the first of two geographically based studies conducted in Delaware, the state that leads the US in TNBC incidence,^44^ we spatially identified TNBC “hot” and “cold” spots.^45^ The proportion of Black breast cancer patients within the hot spots was 10‐fold higher than observed for cold spots, establishing an association between area‐level racial composition and TNBC. However, given the well‐documented higher rates of TNBC observed for Black women, we could not rule out that the TNBC hot spots truly reflected neighborhood effects and not simply geographic areas composed of individuals who were at greater individual risk of TNBC.^46^ In a second multilevel spatial study that adjusted for patient‐level race, we observed that women in predominantly Black census tracts were at greater risk of TNBC, suggesting that neighborhood context contributed to TNBC risk.^47^ We additionally found preliminary support for disparate exposure to specific contextual factors (e.g., alcohol retailer density^48^) within predominantly Black/high‐risk TNBC census tracts that have been shown to influence patient‐level exposure to TNBC risk factors (e.g., unhealthy alcohol use^49^, ^50^).

The objective of this study was to extend this research and conduct a comprehensive assessment of contextual exposures that may help to explain the relationship between neighborhood factors and TNBC risk. We considered all known domains of contextual exposures that have been shown to impact patient‐level exposure to TNBC risk factors, including unhealthy alcohol use, metabolic dysfunction (e.g., diabetes), and reproductive factors (e.g., breastfeeding).^20^, ^26^, ^51^, ^52^, ^53^ In addition, based on emerging evidence of a link between breast cancer and air pollution,^54^, ^55^ we included measures of hazardous environmental exposures. Finally, because adverse neighborhood characteristics often cluster within racially segregated communities,^56^, ^57^, ^58^, ^59^ we evaluated the relationship between cumulative exposure to multiple contextual risk factors and TNBC risk.

## 2 METHODS

### 2.1 Study design, setting, and population

This single‐institution retrospective study was conducted with data from two patient cohorts from New Castle County, Delaware. New Castle County is the catchment area for the Helen F. Graham Cancer Center & Research Institute (HFGCCRI), which is a part of the Christiana Care Health System.

The first patient cohort (“Breast Cancer Cohort”) was composed of breast cancer patients treated at the HFGCCRI. The HFGCCRI provides cancer care to more than 600 analytic breast cancer cases annually, including 85% of all breast cancer cases in New Castle County.^45^ As reported previously, a comparison of this Breast Cancer Cohort with descriptive statistics based on all New Castle County cases provided by the Delaware Cancer registry found that our study population was, in aggregate, representative in terms of patient age, race, subtype, and stage.^45^ Patient records came from the HFGCCRI cancer registry for 3316 adult female New Castle County residents who were diagnosed with invasive breast cancer between 2012 and 2020 (see Figure S1 for flow diagram). This study focused on examining Black‐White disparities; therefore, the population was limited to women who self‐reported their race as either Black or White, regardless of ethnicity. The time frame was selected to maximize the number of cases where the subtype markers necessary for classifying breast cancer cases into TNBC and other subtypes were routinely documented in the cancer registry.

The second patient cohort (“General Hospital Cohort”) was used to generate census tract‐level prevalence estimates of alcohol use disorder and diabetes in New Castle County. Patient records came from electronic health record (EHR) data from the Christiana Care Health System, which provides 88% of all non‐veteran adult acute care in New Castle County.^60^ This cohort consisted of 20,310 unique adult New Castle County residents who were admitted to an inpatient unit between July 1, 2018, and June 30, 2019 (see Figure S2 for flow diagram). To produce the most reliable area‐level prevalence estimates, patients were included in this cohort regardless of admitting diagnosis (i.e., patients with and without a breast cancer diagnosis) or demographics (i.e., men and women of all races/ethnicities). The one‐year time frame was selected to (1) capture any seasonal variation in hospital admissions and (2) use the most recent full‐year of data available that preceded the disruptions imposed by the COVID‐19 pandemic. Prevalence estimates for each diagnosis were generated from the General Hospital Cohort using International Classification of Diseases (ICD) diagnosis codes abstracted from the Christiana Care Health System EHR (codes provided in Supplement). Patient records were geocoded with ArcGIS 10.8^61^ (match rate 98%) and aggregated to their census tract for generating the tract‐level AUD and diabetes prevalence measures. Our previous research has shown that, given the large share of acute care provided by the Christiana Care Health System in New Castle County, prevalence estimates of health behaviors generated from inpatient data show large correlations with available small‐area estimates of risk behaviors provided by the Centers for Disease Control and Prevention (CDC).^62^

This study was reviewed and approved by the Christiana Care Health System Institutional Review Board and was conducted in accordance with the US Common Rule. The need for consent was waived for both patient cohorts.

### 2.2 Measures

Patient measures for the Breast Cancer Cohort included age at diagnosis, race, residential address, and breast cancer subtype markers, which were abstracted from the HFGCCRI cancer registry. Breast cancer cases were classified as “TNBC” when ER, PR, and HER2 were all known negative; all other cases were classified as “Non‐TNBC.”^63^ Patient addresses were geocoded with ArcGIS 10.8,^61^ yielding a 95% match rate. As described elsewhere,^47^ unmatched patients did not significantly differ from matched patients on demographic or clinical characteristics.

A total of 19 census tract‐level measures were initially considered as proxies for each of the four domains of contextual exposures under consideration: unhealthy alcohol use, metabolic dysfunction, breastfeeding, and environmental factors. These were reduced to a final measure for each domain based on both statistical and substantive considerations (see Supplemental text and Tables S1–S3). Tract‐level race data were obtained from the US Census Bureau's American Community Survey.^64^

Tract‐level alcohol use disorder (AUD) prevalence and diabetes prevalence estimates generated from the General Hospital Cohort were selected as the proxy measures for contextual unhealthy alcohol use and metabolic dysfunction, respectively. Tract‐level “percentage of single female‐headed households with children” was selected as the proxy measure for breastfeeding initiation. This measure was obtained from the US Census Bureau's American Community Survey.^64^

Tract‐level Risk Screening Environmental Indicators (RSEI) cancer risk score^65^ was selected as the proxy measure for hazardous environmental exposures using data from the Environmental Protection Agency. The unitless RSEI cancer risk score quantifies the relative risk of exposure to carcinogenic emissions from industrial facilities accounting for the quantity released, chemical toxicity, and fate and transport of chemical compounds. RSEI cancer scores were categorized into quintiles based on their New Castle County distribution to facilitate comparisons.

Finally, an overall cumulative risk score was calculated based on methods used in other studies of cumulative exposures and health.^66^, ^67^ A 0, 1 indicator was assigned for each of the four tract‐level risk factors according to whether it was below or above the median tract‐level value, respectively. The indicators were summed to create a tract‐level cumulative risk score ranging from 0 (best) to 4 (worst).

### 2.3 Statistical analysis

Spatial data management and statistical analyses were performed in the R Statistical Computing Environment.^68^ Descriptive and bivariate statistics were used to compare the TNBC vs. Non‐TNBC groups by individual‐ and census tract‐level characteristics.

Two multilevel logistic regression models were used to examine the odds of TNBC (vs. Non‐TNBC). Both models adjusted for individual (level 1) variables and either included census tract (level 2) variables individually or summarized in the cumulative exposure risk score. Individual‐level variables included age at diagnosis and race. Tract‐level variables included AUD prevalence, diabetes prevalence, percentage single female‐headed households with children, and the RSEI cancer score. A census tract‐level random effect was tested for inclusion in both models to account for the clustering of patients within tracts.

The spatial covariation of the cumulative exposure risk score and TNBC risk was visualized with a boxplot and a choropleth map. Superimposed on the choropleth map are New Castle County TNBC hot and cold spots that were previously detected and described elsewhere.^45^ Briefly, hot and cold spots were identified with the method of spatial intensity, defined as the expected number of cases per area unit.^69^ Spatial intensity was estimated for both TNBC and Not‐TNBC cases separately, and the ratio of their intensities was used to nonparametrically estimate the spatial variation in the odds of TNBC.^70^, ^71^ Tolerance regions with significantly higher or lower odds of TNBC (i.e., hot and cold spots) were identified using the random labeling approach with spatial point patterns of two types.^72^, ^73^

The spatial covariation between the cumulative exposure risk score and census tract racial composition was visualized with a bivariate choropleth map,^74^, ^75^ where each variable was sorted into three quantiles and combined to create a 3 × 3 classification system of low, medium, and high for both measures.

## 3 RESULTS

Of the 3316 cases in the Breast Cancer Cohort, 453 (13.6%) were classified as TNBC and 2863 (86.3%) as Non‐TNBC (Table 1). Relative to Non‐TNBC cases, TNBC cases were younger (60.1 vs. 62.8 years at diagnosis) and a larger percentage of TNBC cases were Black (38.7% vs. 20.0%). TNBC cases lived in census tracts characterized by higher average rates of AUD, diabetes, % single‐female‐headed households with children, and RSEI cancer score risk. Other/unknown race made up 3.9% of the cases and were only presented here for descriptive purposes; as noted above, analyses only included patients who identified as Black or White.

**TABLE 1:** Characteristics of breast cancer patients by subtype in New Castle County, Delaware.

	TNBC (N = 453)	Non‐TNBC (N = 2863)	Total (N = 3316)
Individual characteristics			
Age, mean (SD)	60.1 (14.7)	62.8 (13.2)	62.4 (13.5)
Race, n (%)			
White	274 (59.3%)	2266 (75.9%)	2540 (73.6%)
Black	179 (38.7%)	597 (20.0%)	776 (22.5%)
Other/unknown race(s)	9 (1.9%)	124 (4.2%)	133 (3.9%)
Census tract‐level risk factors			
Alcohol use disorder (AUD) prevalence, mean (SD)	16.9% (6.17%)	16.1% (5.50%)	16.2% (5.60%)
Diabetes prevalence, mean (SD)	36.8% (6.53%)	35.0% (6.74%)	35.3% (6.74%)
% single female‐headed households with children, mean (SD)	13.9% (10.6%)	11.6% (9.27%)	11.9% (9.50%)
RSEI^a^ cancer score quintiles, *n* (%)			
Q1 (lower risk)	51 (11.0%)	467 (15.6%)	518 (15.0%)
Q2	116 (25.1%)	644 (21.6%)	760 (22.0%)
Q3	89 (19.3%)	747 (25.0%)	836 (24.2%)
Q4	103 (22.3%)	615 (20.6%)	718 (20.8%)
Q5 (higher risk)	103 (22.3%)	514 (17.2%)	617 (17.9%)

^a^ Risk Screening Environmental Indicators.

As summarized in Table 2, univariate fixed‐effects analyses estimated that individual‐level characteristics of younger age and Black race and the census tract‐level risk factors of AUD prevalence, diabetes prevalence, percentage female‐headed households with children, higher RSEI cancer scores, and the cumulative exposure risk score were all significantly associated with higher TNBC odds. However, in the first multivariate fixed‐effects model, only the individual characteristics and the second quintile (Q2) of the RSEI cancer score were significantly associated with higher TNBC odds. In the second multivariate fixed‐effects model, considering the tract‐level risk factors summarized into a cumulative exposure risk score as previously described, the individual characteristics and the tract‐level cumulative exposure risk score were significantly associated with greater TNBC odds. Both multivariate models exemplify the disproportionate burden of TNBC among Black women, with more than double the odds of TNBC for Black compared to White patients. In the second model, results show a 10% increase in the odds of TNBC for every one unit increase in cumulative exposure, adjusting for both age and race. When these analyses were attempted with a multivariate mixed‐effects model, it resulted in a singular fit and coefficients could not be estimated (see Table S4). This was likely due to insufficient between‐tract variation to support estimation of a tract‐level random effect, as described in more detail elsewhere.^47^

**TABLE 2:** Odds of triple negative breast cancer (TNBC) by census tract‐level risk factors among Black and White breast cancer patients in New Castle County, Delaware.

	Univariate fixed‐effects, OR, 95% CI	Multivariate fixed‐effects, census tract risk factors AOR, 95% CI	Multivariate fixed‐effects, cumulative risk score AOR, 95% CI
Individual characteristics			
Age^a^	0.93 (0.89, 0.96)**	0.94 (0.91, 0.98)*	0.94 (0.91, 0.98)*
Black race (ref = White)	2.48 (2.01, 3.05)**	2.10 (1.66, 2.65)**	2.17 (1.73, 2.71)**
Census tract‐level risk factors			
Alcohol use disorder (AUD) prevalence^b^	1.12 (1.03, 1.21)*	0.96 (0.86, 1.07)	—
Diabetes prevalence^b^	1.21 (1.13, 1.31)**	1.07 (0.98, 1.17)	—
% single female‐headed households with children^b^	1.12 (1.07, 1.17)**	1.03 (0.96, 1.11)	—
Risk Screening Environmental Indicators (RSEI) cancer score quintiles (ref = Q1)			
Q2	1.77 (1.24, 2.55)*	1.66 (1.16, 2.41)*	—
Q3	1.15 (0.80, 1.68)	1.17 (0.80, 1.72)	—
Q4	1.59 (1.11, 2.30)*	1.35 (0.93, 1.97)	—
Q5 (higher risk)	1.89 (1.32, 2.74)**	1.40 (0.95, 2.08)	—
Cumulative exposure risk score^c^	1.21 (1.12, 1.30)**	—	1.10 (1.01, 1.19)*

^a^ OR and AORs correspond to 5‐year increases.

^b^ OR and AORs correspond to 5% increases.

^c^ Corresponds to sum of 0, 1 indicators for below/above median tract‐level values of AUD prevalence, diabetes prevalence, % single female‐headed households with children, and RSEI cancer score. Treated as a continuous variable for modeling.

* Significant at *p* < 0.05.

** *p* < 0.001.

**FIGURE 1:**
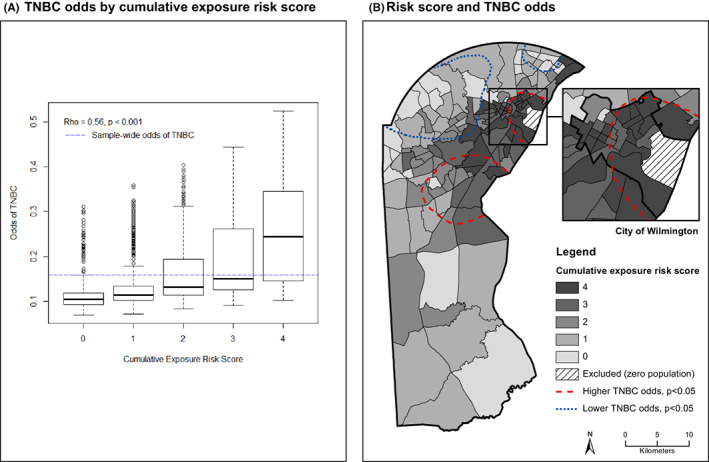
(A) Shows individual‐level odds of triple negative breast cancer (TNBC) by census tract‐level cumulative risk score, derived from model adjusting for age and race. (B) Shows the geographic distribution of census tract cumulative risk scores in New Castle County, Delaware, overlaid with dashed outlines denoting areas with significantly higher or lower odds of TNBC.

Figure 1 visualizes the relationship between cumulative exposure and risk of TNBC. Figure 1A depicts the unadjusted relationship between the cumulative exposure risk score and TNBC odds aggregated to the census tract. The linear relationship between cumulative exposure and odds of TNBC supports treating the cumulative exposure variable as continuous in the regression analysis (Table 2). Figure 1B is a choropleth map that visualizes the tract‐level cumulative exposure risk score in relationship to the TNBC hot and cold spots previously identified.^45^ A visual inspection shows a clear relationship between cumulative risk and TNBC in the Wilmington hot spot, with less of a clear relationship for the southern hot spot. By contrast, the cumulative exposure risk scores are consistently low for both TNBC cold spots.

**FIGURE 2:**
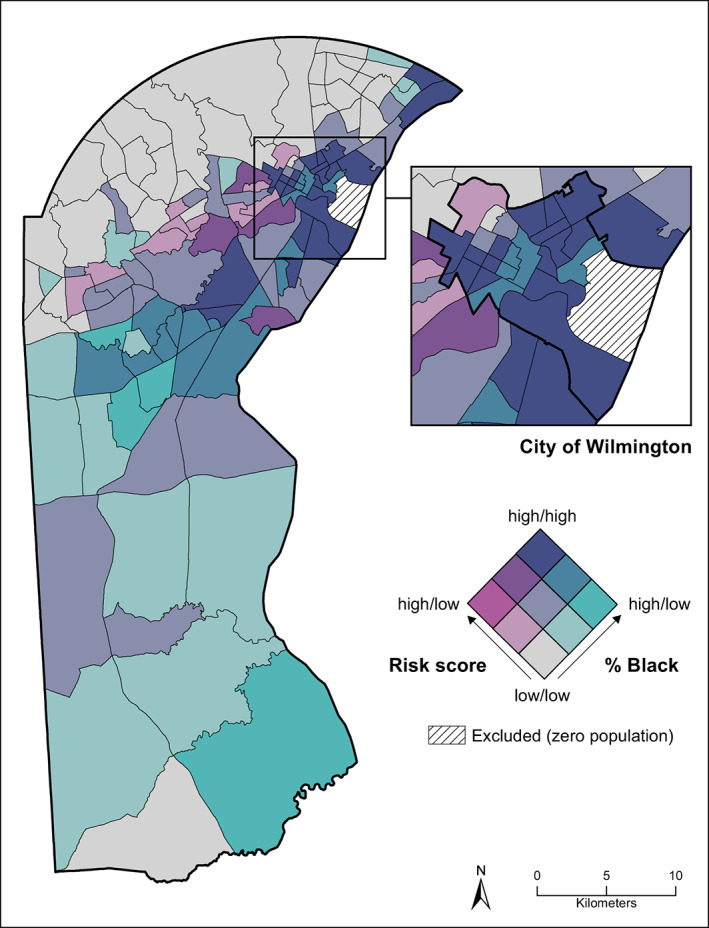
Bivariate choropleth map of census tract‐level cumulative risk scores and percent Black population in New Castle County, Delaware. Color and saturation represent tertials of each variable based on the county distribution. Darker shades of pink correspond to tracts with higher cumulative risk scores, while darker shades of blue correspond to tracts with higher percent Black population. Tracts in the darkest shade of purple are in the top tertial for each variable.

Figure 2 is a bivariate choropleth map that visualizes the spatial covariation of the tract‐level cumulative exposure risk score and tract‐level percentage Black residents. Areas shaded in dark purple represent tracts that are high in cumulative risk and are composed predominantly of Black residents, which can be found in and around the city of Wilmington and the northeastern part of the county. Areas shaded in gray represent tracts that are low in cumulative risk and are composed predominantly of White residents, which are mostly found in the northwest region of the county. The remaining shades represent different combinations of risk and racial composition.

## 4 DISCUSSION

In a cohort of breast cancer patients from New Castle County, Delaware, the state with the highest rates of TNBC in the US,^44^ we found evidence of associations between multiple domains of contextual community and environmental exposures and greater odds of TNBC relative to other invasive subtypes of breast cancer. More specifically, in multilevel analyses that adjusted for individual‐level age and race, we observed univariate associations with census tract‐level measures of AUD prevalence, diabetes prevalence, percentage single female‐headed households with children (as a proxy for breastfeeding rates), and hazardous environmental exposures. In a multivariate analysis that included individual‐level and contextual risk factors, only age and race were significantly associated with TNBC odds. However, when the tract‐level contextual risk factors were combined into a tract‐level cumulative exposure risk score, it was significantly associated with TNBC odds in a multilevel model. For every additional contextual risk factor, the odds of TNBC increased by 10%. To our knowledge, this is the first report to examine the relationship between cumulative exposure to contextual risk factors and TNBC risk.

Estimating cumulative exposure to contextual risk factors is consistent with a *causal architecture* approach described by Keyes and Galea (2017).^76^ As they argue, traditional risk factor approaches are implicitly founded on reductionist biomedical models of illness. More contemporary models of cancer carcinogenesis, by contrast, emphasize multilevel frameworks that account for the context in which disease originates.^43^ The importance of context was particularly apparent in Wilmington, Delaware, previously identified as a TNBC hot spot.^45^ Wilmington has been cataloged as a “small industrial‐legacy city,” characterized by predominantly Black populations, high poverty rates, and reduced life expectancy.^77^ Wilmington and similar communities also have higher densities of liquor stores and limited healthy food options, which contribute to unhealthy alcohol use and metabolic dysfunction.^48^, ^78^, ^79^ These same contextual factors have been linked to a reduced likelihood of breastfeeding.^80^, ^81^, ^82^ Finally, as reviewed by Bentlyewski and Juhn,^83^ an individual's race is the most important predictor of living in proximity to a toxic site, which is consistent with our findings (see Table S2) and an independent report on toxic exposures in New Castle County, Delaware.^84^ By stark contrast, the more affluent, predominantly White northwestern section of New Castle County was uniformly low in cumulative risk. Thus, high levels of multicollinearity within racially segregated groups may explain the absence of significant contextual effects in the multivariate model that included discrete (vs. cumulative) tract‐level risk factors. For both statistical and ecological reasons, attempts to evaluate risk factors independent of context may lead to biased results and undermine efforts to improve primary and secondary TNBC prevention.^85^, ^86^

This study has several important limitations. First, the single‐site, cross‐sectional design precludes drawing causal and generalizable inferences. Second, we did not have access to residential histories or patient‐level measures of relevant exposures (e.g., alcohol use), which may introduce potential exposure misclassification. Third, while our prior research suggests that the area‐level exposure estimates generated from EHR data are strongly correlated with small‐area estimates provided by the CDC,^62^ it is important to note that small‐area estimates are not available for all exposures of interest (e.g., AUD) and geographic areas and therefore precludes our ability to fully assess for bias in our estimates. Fourth, while EHR‐based prevalence estimates for diabetes have good sensitivity and specificity,^87^, ^88^ AUD often goes undetected in non‐psychiatric hospital settings for patients with less severe forms of the disorder (i.e., not exhibiting symptoms of acute withdrawal).^89^ This raises the strong possibility that our AUD prevalence rates represent underestimates. Nevertheless, the distribution of alcohol consumption has been shown to maintain a relatively stable shape across populations such that rates of the most severe forms of AUD are proportional to rates of less severe forms of AUD and unhealthy alcohol use more generally.^90^, ^91^ Thus, while the EHR‐generated absolute AUD prevalence rates may represent underestimates, there is much less concern this would impact the ordering of area‐level rates or the relationship between AUD prevalence and TNBC risk. Fifth, it is also possible that recent environmental exposure data and industrial emissions in proximity to a current home address may not represent latent exposures patterns, which are particularly important for cancer outcomes. Given these limitations, future research should replicate and validate these findings across cohorts and geographic regions with patient‐level data on lifecourse exposures to further evaluate the cumulative, multilevel influences on TNBC risk. In the interim, however, the findings from this study can be broadly hypothesis‐generating and guide local efforts to target racial disparities in breast cancer in a state that has notably elevated rates of TNBC.

Beyond the replication and validation of our findings with similar methods, we wish to highlight the example and methodological recommendations offered by a few key researchers to further advance research that considers structural racism, residential segregation, and health disparities.^92^, ^93^, ^94^, ^95^ Riley^85^, ^92^ has argued that social stratification—more than spatial stratification—impacts exposure to risk factors. Homan, Brown, and King^94^ draw on the intersectionality framework^96^ and elaborate on how overlapping forms of discrimination based on race, sex, gender, and class can impact systems of exposure. Thus, Black and White women living within the same census tract likely experience differential exposure to TNBC risk factors. These differences in exposure based on social stratification are the direct result of structurally racist policies and institutional actors, as argued by Sewell and others.^93^ To better study these processes, Riley^92^ recommends more consideration be given to the spatial and temporal units of analysis. For example, the exposure to alcohol retailers or hazardous pollutants may vary considerably within a census tract and over time. Conversely, state‐level criminal justice policies may be more relevant to family structure in minority communities (e.g., single female‐headed households). Ultimately, to advance this field of study and develop interventions that close cancer disparities, the research question should be informed by our understanding of structural racism, which in turn should inform the methods.

In conclusion, the results from this study extend prior research that has documented a relationship between general neighborhood characteristics (i.e., SES, segregation) and TNBC risk^28^, ^29^, ^47^ by considering the cumulative exposure to contextual factors as a potential mediator of this relationship. Building on this finding, multiple lines of future research can inform the development of multilevel interventions targeting TNBC risk within a broader health equity approach. First, spatial methods can guide more geographically targeted outreach to communities at greatest risk for TNBC, a key component of cancer control and prevention.^97^ Unlike other countries that have instituted organized breast cancer screening programs, which systematically test all women in a defined group, the US approach to screening is more opportunistic and relies on clinician or self‐referral to screening mammography.^98^ However, the evidence suggests that organized approaches can reduce disparities in screening utilization.^99^ The methods employed in this study can help to define high‐risk geographic areas for organized screening programs, which can then be evaluated as a catchment area‐based approach for reducing cancer health disparities. Second, existing TNBC risk prediction models have performed poorly in formal evaluations.^25^, ^100^ Given the consistent link between area‐level factors and TNBC risk, new research is needed to evaluate whether contextual variables can improve TNBC risk prediction.^101^ Better risk stratification could help to engage at‐risk Black women in early detection screening programs at a younger age (i.e., before 50), which could lead to significant reductions in breast cancer disparities.^102^ Improved risk stratification could also inform the implementation of risk‐reduction programs, such as targeting unhealthy alcohol use in primary care settings.^103^ Finally, delineating the relationship between cumulative area‐level exposures and TNBC risk can support policymakers' efforts to develop place‐based interventions designed to mitigate racial disparities in breast cancer mortality communitywide.

## AUTHOR CONTRIBUTIONS

**Scott Siegel:** Conceptualization (lead); project administration (lead); resources (lead); writing – original draft (lead). **Madeline Brooks:** Data curation (lead); formal analysis (lead); visualization (lead); writing – original draft (equal); writing – review and editing (equal). **Jesse Berman:** Conceptualization (equal); methodology (equal); writing – review and editing (supporting). **Shannon Lynch:** Conceptualization (equal); writing – review and editing (equal). **Jennifer Sims‐Mourtada:** Conceptualization (equal); data curation (equal); writing – review and editing (supporting). **Zachary Schug:** Conceptualization (supporting); writing – review and editing (supporting). **Frank Curriero:** Conceptualization (equal); methodology (lead); supervision (equal); writing – original draft (supporting); writing – review and editing (equal).

## FUNDING INFORMATION

This project was supported by NIGMS (P20 GM103446) from the NIH and the State of Delaware (to S.D. Siegel).

## CONFLICT OF INTEREST STATEMENT

The authors declare that they have no competing interests.

## Supporting information


Figure S1–S2:

Table S1–S4:

## Data Availability

Datasets generated for this study from the HFGCCRI cancer registry or the Christiana Care Health System EHR are not publicly available because they contain protected health information but may be made available in a deidentified format from the corresponding author on reasonable request. Census tract datasets generated for this study based on publicly available sources are available from the corresponding author on reasonable request.
