# Supplementary material for: Neighborhood factors and triple negative breast cancer: The role of cumulative exposure to area‐level risk factors
Source: Cancer Med. 2023 Mar 14;12(10):11760–72. doi: 10.1002/cam4.5808 (PMC10242317; doi:10.1002/cam4.5808)

Supplement Figure 1. Breast Cancer Cohort Flow Diagram

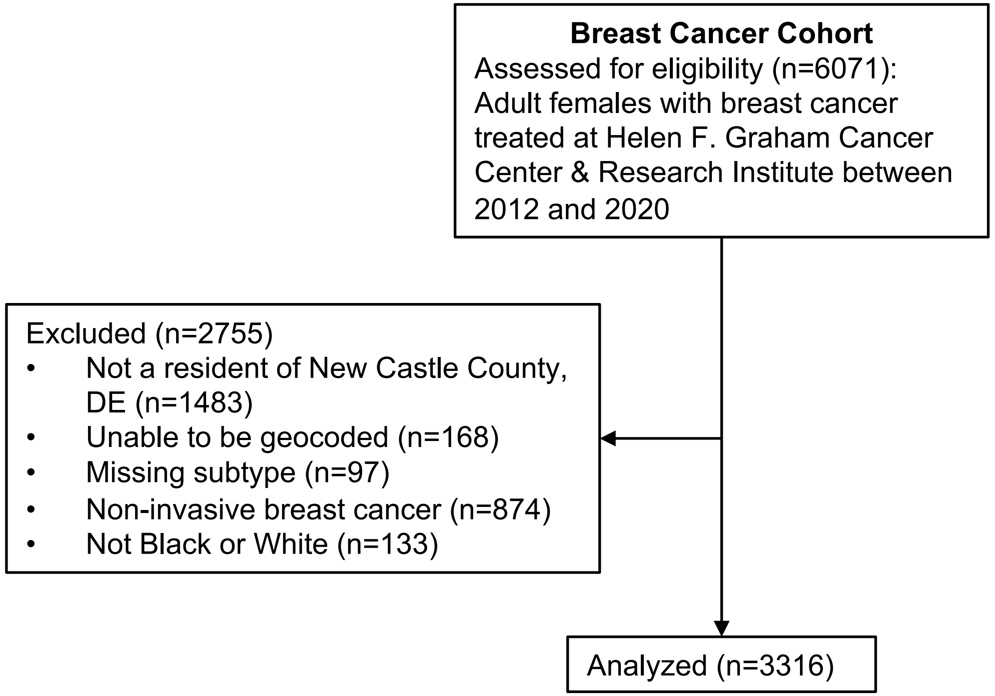

Supplement Figure 2. General Hospital Cohort Flow Diagram

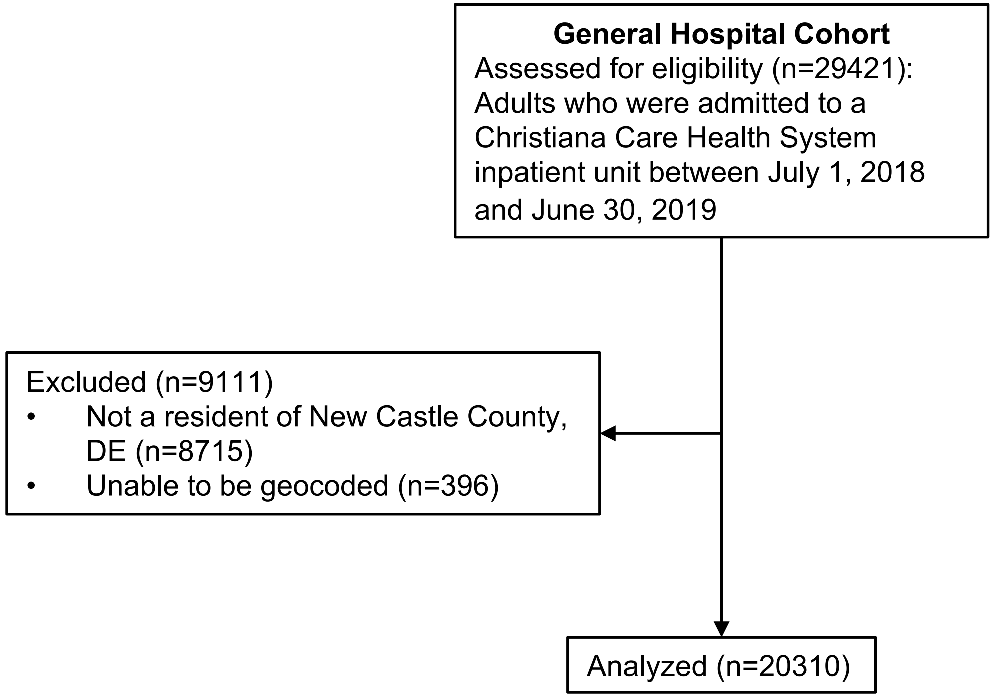

Supplement: Supplementary file 1 — Figure S1–S2: [file CAM4-12-11760-s002.pdf]
