# Supplementary material for: Neighborhood factors and triple negative breast cancer: The role of cumulative exposure to area‐level risk factors
Source: Cancer Med. 2023 Mar 14;12(10):11760–72. doi: 10.1002/cam4.5808 (PMC10242317; doi:10.1002/cam4.5808)
Supplement: Supplementary file 2 — Table S1–S4: [file CAM4-12-11760-s001.docx]

**Supplemental Section**

**METHODS: Candidate Contextual Risk Factor Measures**

Candidate contextual risk factor measures were considered for all known domains of area-level exposures that have been shown to impact patient-level exposure to TNBC risk factors. As reviewed in the main text, these domains included unhealthy alcohol use, metabolic dysfunction, reproductive factors, and hazardous environmental exposures. Consistent with the recommendation provided by Riley,^1^ we considered multiple data sources for each domain.

Four tract-level measures were considered for unhealthy alcohol use: alcohol retailers (aggregated count and population adjusted), alcohol retailer visit rate, and alcohol use disorder (AUD) prevalence. Alcohol retailer data were extracted from a public Delaware business license database that was current as of April 17, 2019.^2^ Prior research has found a more consistent link between unhealthy alcohol use and exposure to off-premise alcohol retailers (e.g., liquor stores) relative to on-premise retailers (e.g., bars).^3^ Therefore, only licensed establishments that sold alcoholic beverages for offsite consumption were included. All alcohol retail locations were geocoded with ArcGIS 10.8 with a 100% match rate. Aggregated count and population-adjusted counts (per 1000 census tract residents) were generated for each census tract. Alcohol retailer visit rates were calculated with anonymized mobile phone location data provided freely to researchers by the company SafeGraph, consistent with the methods described by Chang and colleagues^4^ and SafeGraph technical guidance.^5^ Briefly, alcohol retailer visits were captured based on global positioning system (GPS) point location data produced by the mobile phone relative to alcohol retailer “footprints.” The alcohol retailer visit rate represents the total number of visitors from a census tract divided by the total number of mobile devices inferred to belong to a resident from that same census tract. Consistent with our methods for generating alcohol retailer counts, only visits to off-premise alcohol retailers (those categorized by NAICS code 445310) were included. Prior research has shown that alcohol retailer visit rates are correlated with alcohol sales^6^ and contribute unique information over and above alcohol retailer counts to the prediction of domestic violence, a serious public health issue strongly linked to unhealthy alcohol use.^4^ The measurement of AUD prevalence is described in the main text.

Six tract-level measures were considered for contextual factors that could impact patient-level metabolic dysfunction: fast food retailer counts (aggregated counts and population-adjusted), fast food retailer visit rate, the modified retail food environment index (mRFEI), obesity prevalence, and diabetes prevalence. Fast food retailer data were obtained from SICCODE.com,^7^ utilizing the North American Industry Classification System (NAICS) code 722513, consistent with established approaches.^8^ Census tract counts were generated with the same approach used for alcohol retailers. The fast-food retailer visit rate was also calculated with the same approach used for the alcohol retailer visit rate described above. The mRFEI^8^ represents the proportion of food retailers within a half-mile of census tract boundaries that are categorized as ‘healthy.’ Healthy food retailers are defined as supermarkets, grocery stores, and other stores that sell produce (NAICS codes: 445110, 44523, and 452910, respectively); ‘less healthy’ food retailers are defined as fast-food restaurants, convenience stores, and other small grocers unlikely to sell produce (NAICS codes: 722211, 445120, 445110, respectively). Obesity and diabetes prevalence rates were calculated from the General Hospital Cohort with the same approach used for AUD prevalence, utilizing ICD codes abstracted for diabetes and the admitting height and weight data for obesity from the Christiana Care Health System EHR. Obesity was defined as a body mass index > 30, consistent with established practices.^9^

Four tract-level measures were considered for breastfeeding: birth rates for mothers aged 15-19 or aged 15-50, the percentage of women employed by sector (management, business, science, and arts occupations; service occupations), and the percentage of single female-headed households with children. Absent a more direct measure of breastfeeding initiation, these measures were considered because they were available at the tract-level and represent structural or demographic factors that have been linked to breastfeeding rates.^10,11^ All four measures were obtained from the U.S. Census Bureau’s American Community Survey 5-year estimates for 2014-2018.^12^

Four tract-level measures were considered for hazardous environmental exposures: ambient annual average fine particulate air pollution (PM_2.5_) averaged across 2006-2015 provided by the Center for Air, Climate, and Energy Solutions (CACES)^13^; Toxics Release Inventory (TRI) site locations^14^ from the Environmental Protection Agency (EPA; aggregated counts and aggregated intensity); and the EPA’s Risk Screening Environmental Indicators (RSEI) cancer risk score.^15^ TRI sites manufacture, process, or use toxic chemicals that may impact human health, voluntarily reporting to the Environmental Protection Agency the identity and quantity of total substances released each year. The aggregated intensity version of the TRI measure was generated using methods described in more detail elsewhere.^16^ Briefly, this approach uses kernel density estimation to generate spatially continuous expected counts of TRI sites per unit area (1 KM bandwidth) before aggregating these results to census tracts, producing proximity-weighted, tract-level TRI counts that are initially independent of tract boundaries. Additional details on the RSEI cancer risk score are provided in the main text.

**RESULTS: Selection of Contextual Risk Factor Measures**

The selection of a final measure for each domain was based on both statistical and substantive considerations. Consistent with the first step in a forward selection statistical procedure^17^ and neighborhood-wide association studies (NWAS),^18^ candidate proxy contextual risk factors were first compared by breast cancer subtype (TNBC vs. Non-TNBC; Supplemental Table 1). The census tract variables for alcohol use were all significantly higher in the TNBC (vs. Non-TNBC) group. Among the metabolic variables, only obesity and diabetes prevalence were significantly higher in the TNBC group, with no differences for the fast-food retailer variables or the mRFEI. For the reproductive variables, no differences were observed for birth rates. However, TNBC patients lived in census tracts with significantly lower average percentages of women working in management and related sectors and significantly higher percentages of women working in service occupations. Finally, TNBC patients lived in census tracts with a significantly higher percentage of female-headed households with children. For the environmental variables, neither CACES PM2.5 (10-year annual average) nor TRI sites (aggregated counts) were significantly different between groups. However, when TRI exposure was estimated with aggregated intensity, values were significantly higher for the TNBC group. Likewise, a larger percentage of TNBC cases resided in census tracts in the highest-risk quintile for the RSEI cancer score. Within each domain, variables that were associated with TNBC status on a univariate basis were significantly correlated with each other, necessitating further reduction of our candidate variables to prevent multicollinearity in final models.

Next, the association between contextual risk factor variables and tract-level percentage Black population was examined (Supplemental Table 2). The variables in each category with the strongest correlations were AUD prevalence, diabetes prevalence, % single female-headed households with children, and the RSEI cancer score. As reviewed above, each of these variables were also significantly different by TNBC group status in the expected direction.

Additional substantive support for the selection of these four proxy variables was considered. In the case of the alcohol and metabolic variables, it can be argued that disease prevalence is the most valid measure relevant to patient-level exposure. For example, while exposure to a greater number of alcohol retailers has been consistently associated with higher population-level rates of unhealthy alcohol use,^3^ the contextual exposure of greatest interest is the one most strongly predictive of patient-level exposure. Higher rates of tract-level AUD prevalence are conceptualized to be a function of the alcohol retailer environment and other contextual variables (e.g., SES), and potentially a more valid contextual indicator of exposures that contribute to patient-level unhealthy alcohol use. Similarly, higher rates of tract-level diabetes prevalence are conceptualized to be a function of the food environment and other contextual variables, and potentially a more valid contextual indicator of exposures that contribute to patient-level rates of diabetes.

For the reproductive variables, other research has consistently found that single female-headed household status is strongly predictive of breastfeeding rates at the individual-level.^10,11^ We also examined this association at the county level by examining the correlations between the candidate reproductive variables and breastfeeding initiation rates for 2018-2019 provided by the Centers for Disease Control and Prevention and the National Vital Statistics System, which are available for all states except California and Michigan.^19^ At the county level, percentage single female-headed households with children again emerged as the strongest correlation (Supplemental Table 3)

Finally, for the environmental variables, we considered that the RSEI score accounts for the quantity, toxicity, and fate and transport of chemical release and the potential for population-level exposure.^15^ By contrast, the aggregated intensity TRI measure is a proximity measure and does not directly incorporate information about chemical exposure.

In our final analysis, we chose the following tract-level contextual variables based on their relationship to TNBC status, tract-level racial composition, and additional substantive considerations: AUD prevalence, diabetes prevalence, % single female-headed households with children, and RSEI cancer score.

Supplemental Table 1. Census tract-level characteristics among Black and White breast cancer patients by subtype in New Castle County, Delaware

| Census Tract Characteristics | TNBC  (N=453) | Non-TNBC  (N=2863) | Total  (N=3316) |
| --- | --- | --- | --- |
| **Alcohol variables** | | | |
| Alcohol retailers, mean (SD)** | 1.49 (1.33) | 1.27 (1.22) | 1.30 (1.24) |
| Alcohol retailers/1K people, mean (SD)* | 0.30 (0.30) | 0.27 (0.28) | 0.27 (0.29) |
| Alcohol retailer visits/1K people, mean (SD) | 142 (61.5) | 137 (54.2) | 138 (55.3) |
| Alcohol use disorder (AUD) prevalence, mean (SD)* | 16.9 (6.19) | 16.1 (5.56) | 16.3 (5.65) |
| **Metabolic variables** | | | |
| Fast food retailers, mean (SD) | 2.28 (3.39) | 2.21 (3.51) | 2.22 (3.49) |
| Fast food retailers/1K people, mean (SD) | 0.40 (0.49) | 0.40 (0.59) | 0.40 (0.58) |
| Fast food retailer visits/1K people, mean (SD) | 857 (253) | 869 (256) | 867 (255) |
| Modified retail food environment index (mRFEI), mean (SD) | 14.2 (10.5) | 15.3 (11.1) | 15.2 (11.1) |
| Obesity prevalence, mean (SD)** | 42.2 (6.26) | 40.9 (7.04) | 41.1 (6.95) |
| Diabetes prevalence, mean (SD)** | 36.8 (6.51) | 35.1 (6.74) | 35.3 (6.73) |
| **Reproductive variables** | | | |
| Births/1K ages 15-19, mean (SD) | 8.29 (43.6) | 7.42 (38.1) | 7.54 (38.9) |
| Births/1K ages 15-50, mean (SD) | 40.7 (30.1) | 40.4 (29.5) | 40.4 (29.6) |
| % women employed in management, business, science, and arts occupations, mean (SD)** | 46.8 (12.9) | 49.3 (12.7) | 49.0 (12.7) |
| % women employed in service occupations, mean (SD)** | 18.6 (7.69) | 17.2 (7.38) | 17.4 (7.44) |
| % single female-headed households with children, mean (SD)** | 14.0 (10.7) | 11.7 (9.34) | 12.1 (9.57) |
| **Environmental variables** | | | |
| CACES^a^ PM2.5 ug/m^3^ (2006-2015 average), mean (SD) | 10.8 (0.57) | 10.8 (0.61) | 10.8 (0.60) |
| Toxics Release Inventory (TRI) sites, mean (SD) | 0.31 (0.73) | 0.27 (0.72) | 0.28 (0.72) |
| TRI sites aggregated intensity (1 KM bandwidth), mean (SD)** | 0.33 (0.62) | 0.27 (0.58) | 0.28 (0.58) |
| Risk Screening Environmental Indicators (RSEI) cancer score  quintiles, n (%)* | | | |
| Q1 (lower risk) | 48 (10.6) | 449 (15.7) | 497 (15.0) |
| Q2 | 116 (25.6) | 614 (21.4) | 730 (22.0) |
| Q3 | 89 (19.6) | 705 (24.6) | 794 (23.9) |
| Q4 | 98 (21.6) | 594 (20.7) | 692 (20.9) |
| Q5 (higher risk) | 102 (22.5) | 501 (17.5) | 603 (18.2) |

*Significant at p<0.05, **p<0.001

^a^Center for Air, Climate, and Energy Solutions

Supplemental Table 2. Correlations of census tract-level risk factors and percent Black population in New Castle County, Delaware

|  | Census tract % Black population |
| --- | --- |
| **Alcohol variables** | |
| Alcohol retailers | 0.20 (p=0.020)* |
| Alcohol retailers/1K people | 0.17 (p=0.047)* |
| Alcohol retailer visits/1K people | 0.32 (p<0.001)** |
| *Alcohol use disorder (AUD) prevalence* | 0.41 (p<0.001)** |
| **Metabolic variables** | |
| Fast food retailers | 0.08 (0.366) |
| Fast food retailers/1K people | 0.08 (0.357) |
| Fast food retailer visits/1K people | -0.25 (p=0.004)* |
| Modified retail food environment index (mRFEI) | -0.34 (p<0.001)** |
| Obesity prevalence | 0.54 (p<0.001)** |
| *Diabetes prevalence* | 0.63 (p<0.001)** |
| **Reproductive variables** | |
| Births/1K ages 15-19 | 0.23 (p=0.008)* |
| Births/1K ages 15-50 | 0.12 (p=0.161) |
| % women employed in management, business, science, and arts occupations | -0.56 (p<0.001)** |
| % women employed in service occupations | 0.57 (p<0.001)** |
| *% single female-headed households with children* | 0.72 (p<0.001)** |
| **Environmental variables** | |
| CACES^a^ PM2.5 ug/m^3^ (2006-2015 average) | 0.18 (0.039)* |
| Toxics Release Inventory (TRI) sites | 0.16 (p=0.070) |
| TRI sites aggregated intensity (1 KM bandwidth) | 0.38 (p<0.001)** |
| *Risk Screening Environmental Indicators (RSEI) cancer score* | 0.46 (p<0.001) |

*Significant at p<0.05, **p<0.001

^a^Center for Air, Climate, and Energy Solutions

Italic text denotes variables selected for final model.

Supplemental Table 3. County-level reproductive variable correlations with breastfeeding initiation rates, 2018-2019

|  | Births/1K, ages 15-50 | Births/1K, ages 15-19 | % women employed in service occupations | % women employed in MBSA occupations | % single female-headed households with children | Breastfeeding initiation rate |
| --- | --- | --- | --- | --- | --- | --- |
| Births/1K, ages 15-50 | -- | 0.17** | 0.10** | -0.14** | 0.00 | 0.03 |
| Births/1K, ages 15-19 | -- | -- | 0.04* | -0.10** | 0.23** | -0.19** |
| % women employed in service occupations | -- | -- | -- | -0.44** | 0.04 | -0.09** |
| % women employed in MBSA^a^ occupations | -- | -- | -- | -- | -0.13** | 0.30** |
| % single female-headed households with children | -- | -- | -- | -- | -- | -0.47** |
| Breastfeeding initiation rate | -- | -- | -- | -- | -- | -- |

*Significant at p<0.05, **p<0.001.

^a^Management, business, science, and arts occupations.

Supplemental Table 4. Mixed-effects models and census tract random effect variance

|  | No covariates | Univariate mixed-effects | | Multivariate mixed-effects, census tract risk factors | | Multivariate mixed-effects, continuous risk score | | Multivariate mixed-effects, categorical risk score | |
| --- | --- | --- | --- | --- | --- | --- | --- | --- | --- |
|  | Random effect variance, ICC | OR, 95% CI | Random effect variance, ICC | AOR, 95% CI | Random effect variance, ICC | AOR, 95% CI | Random effect variance, ICC | AOR, 95% CI | Random effect variance, ICC |
| Age | 0.092 (p=0.014)*  ICC=2.7% | 0.93 (0.89, 0.96)** | 0.079 (p=0.022)*  ICC=2.3% | X | X | X | X | X | X |
| Black race (ref=White) |  | 2.48 (2.01, 3.06)** | 0.002 (p=0.433)  ICC=0.1% | X |  | X |  | X |  |
| Alcohol use disorder (AUD) prevalence^b^ |  | 1.12 (1.02, 1.23)* | 0.078 (p=0.058)  ICC=2.3% | X |  | -- |  | -- |  |
| Diabetes prevalence^b^ |  | 1.22 (1.12, 1.32)** | 0.031 (p=0.317)  ICC=0.9% | X |  | -- |  | -- |  |
| % single female-headed households with children^b^ |  | X | X | X |  | -- |  | -- |  |
| Risk Screening Environmental Indicators  (RSEI) cancer score quintiles (ref=Q1) |  |  | | -- |  | -- |  | -- |  |
| Q2 |  | 1.75 (1.20, 2.55)* | 0.035 (p=0.091)  ICC=1.1% | X |  | -- |  | -- |  |
| Q3 |  | 1.15 (0.78, 1.70) |  | X |  | -- |  | -- |  |
| Q4 |  | 1.59 (1.09, 2.34)* |  | X |  | -- |  | -- |  |
| Q5 |  | 1.90 (1.29, 2.79)* |  | X |  | -- |  | -- |  |
| Cumulative exposure risk score^c^ (continuous) |  | 1.21 (1.12, 1.32)** | 0.043 (p=0.102)  ICC=1.3% | -- |  | X |  | -- |  |
| Cumulative exposure risk score^c^ (categorical) (ref=0) |  |  | | X |  | -- |  | -- |  |
| 1 |  | 1.00 (0.70, 1.44) | 0.038 (p=0.095)  ICC=1.1% | -- |  | -- |  |  |  |
| 2 |  | 1.39 (0.97, 2.00) |  | -- |  | -- |  |  |  |
| 2 |  | 1.40 (0.93, 2.08) |  | -- |  | -- |  |  |  |
| 4 |  | 2.07 (1.43, 3.01)** |  | -- |  | -- |  |  |  |

**ICD Codes**

**The following codes were used to generate EHR-based area-level prevalence estimates for AUD, diabetes, and obesity.**

**Alcohol Use Disorder (AUD)**

- ICD-9-CM: 291, 303, 305, 357, 425, 535, 571, 980
- ICD-10-CM: E52, F10, G62, I42, K70, T51, V11, Z71

**Diabetes**

- ICD-9-CM: 250
- ICD-10-CM: E10, E11, E13

**Obesity**

- ICD-9-CM:278
- ICD-10-CM: E66

**References**

1. Riley AR. Neighborhood Disadvantage, Residential Segregation, and Beyond—Lessons for Studying Structural Racism and Health. *J Racial Ethn Heal Disparities*. 2018;5(2):357-365. doi:10.1007/s40615-017-0378-5

2. Delaware Division of Revenue. Delaware business licenses. Delaware Open Data. Published 2019. Accessed April 17, 2019. https://revenue.delaware.gov/business-license-search/

3. Gmel G, Holmes J, Studer J. Are alcohol outlet densities strongly associated with alcohol-related outcomes? A critical review of recent evidence. *Drug Alcohol Rev*. 2016;35(1):40-54. doi:10.1111/dar.12304

4. Chang T, Hu Y, Taylor D, Quigley BM. The role of alcohol outlet visits derived from mobile phone location data in enhancing domestic violence prediction at the neighborhood level. *Health Place*. 2022;73(August 2021):102736. doi:10.1016/j.healthplace.2021.102736

5. SafeGraph. *Determining Points of Interest Visits from Location Data: A Technical Guide to Visit Attribution*.; 2021.

6. Hu Y, Quigley BM, Taylor D. Human mobility data and machine learning reveal geographic differences in alcohol sales and alcohol outlet visits across U.S. states during COVID-19. *PLoS One*. 2021;16(12 December):1-27. doi:10.1371/journal.pone.0255757

7. SIC Code & NAICS Code Business Lists. NAICS food retailer data [Data file]. SICCODE.com

8. Centers for Disease Control and Prevention. *Census Tract Level State Maps of the Modified Retail Food Environment Index (MREFI)*.; 2011. Accessed July 9, 2021. https://www.cdc.gov/obesity/downloads/census-tract-level-state-maps-mrfei_TAG508.pdf

9. Moyer VA. Screening for and Management of Obesity in Adults: U.S. Preventive Services Task Force Recommendation. *Ann Intern Med*. 2012;157(5). doi:10.7326/0003-4819-157-5-201209040-00482

10. Odar Stough C, Khalsa AS, Nabors LA, Merianos AL, Peugh J. Predictors of Exclusive Breastfeeding for 6 Months in a National Sample of US Children. *Am J Heal Promot*. 2019;33(1):48-56. doi:10.1177/0890117118774208

11. Cohen SS, Alexander DD, Krebs NF, Young BE, Cabana MD, Erdmann P, Hays NP, et al. Factors Associated with Breastfeeding Initiation and Continuation: A Meta-Analysis. *J Pediatr*. 2018;203:190-196.e21. doi:10.1016/j.jpeds.2018.08.008

12. U.S. Census Bureau. 2014-2018 American Community Survey 5-Year Estimates. Published 2020. https://www.census.gov/programs-surveys/acs/technical-documentation/table-and-geography-changes/2018/5-year.html

13. Saha PK, Hankey S, Marshall JD, Robinson AL, Presto AA. High-Spatial-Resolution Estimates of Ultrafine Particle Concentrations across the Continental United States. *Environ Sci {\&} Technol*. 2021;55(15):10320-10331. doi:10.1021/acs.est.1c03237

14. United States Environmental Protection Agency. TRI Basic Data Files: Calendar Years 1987- Present. *Toxics Release Invent Progr*. Published online 2022. Accessed March 30, 2022. https://www.epa.gov/toxics-release-inventory-tri-program/tri-basic-data-files-calendar-years-1987-present

15. United States Environmental Protection Agency. *EPA’s Risk-Screening Environmental Indicators (RSEI) Methodology: RSEI Version 2.3.10*.; 2022. https://www.epa.gov/system/files/documents/2022-06/RSEI

16. Brooks MM, Siegel SD, Corrigan AE, Curriero FC. Aggregated spatial intensity as a method for estimating point-level exposures within area-level units: The case of tobacco retailer exposure in census tracts. *Spat Spatiotemporal Epidemiol*. 2022;41. doi:10.1016/j.sste.2022.100482

17. Blanchet FG, Legendre P, Borcard D. FORWARD SELECTION OF EXPLANATORY VARIABLES. *Ecology*. 2008;89(9):2623-2632. doi:10.1890/07-0986.1

18. Lynch SM, Mitra N, Ross M, Newcomb C, Dailey K, Jackson T, Zeigler-Johnson CM, et al. A Neighborhood-Wide Association Study (NWAS): Example of prostate cancer aggressiveness. *PLoS One*. 2017;12(3):1-13. doi:10.1371/journal.pone.0174548

19. Breastfeeding Initiation Rates by County. https://www.cdc.gov/breastfeeding/data/county/breastfeeding-initiation-rates.html
